# Atomic Layer Deposition of Ti_*x*_Fe_2–*x*_O_3_ Photoanodes and Photocurrent Response Optimization Using the Response Surface Methodology

**DOI:** 10.1021/acsomega.5c01360

**Published:** 2025-04-05

**Authors:** Anjan Deb, Anton Vihervaara, Georgi Popov, Mykhailo Chundak, Ahmed O. Abdelaal, Hugo L. S. Santos, Mikko J. Heikkilä, Marianna Kemell, Pedro H. C. Camargo, Mikko Ritala, Matti Putkonen

**Affiliations:** Department of Chemistry, University of Helsinki, P.O. Box 55, FI-00014 Helsinki, Finland

## Abstract

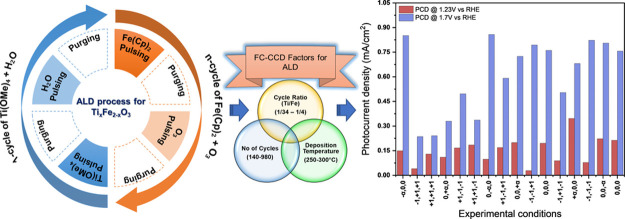

Hematite (Fe_2_O_3_) is a promising visible-light-active semiconductor material for photoelectrocatalytic applications; however, it has yet to achieve its theoretical maximum efficiency. Researchers globally are making significant efforts to enhance its performance and surpass the current efficiency limitations. Here, we report the photoelectrocatalytic performance of Ti_*x*_Fe_2–*x*_O_3_ films deposited by atomic layer deposition (ALD) using FeCp_2_ and Ti(OMe)_4_ as precursors. The response surface methodology (RSM) with a face-centered central composite design (FC-CCD) was used to model and optimize the photocurrent response of Ti_*x*_Fe_2–*x*_O_3_ thin film photoanodes. Deposition parameters, including the cycle ratio of TiO_2_ to Fe_2_O_3_, total number of ALD cycles, and deposition temperature, were selected as independent variables, while the photocurrent density (PCD) at 1.23 and 1.70 V vs RHE was used as the response variable. Thin film depositions were carried out according to the FC-CCD design matrix, followed by postannealing at 500 °C for 1 h in air. The films were then evaluated for their photocurrent response using a photoelectrochemical cell under standard AM 1.5G illumination, 100 mW/cm^2^. The experimental photocurrent responses were fitted to a second-order polynomial equation, resulting in the development of a mathematical model that establishes a relationship between the deposition parameters and PCD of Ti_*x*_Fe_2–*x*_O_3_ photoanode. Analysis of model parameters revealed that film thickness and dopant concentration are the most significant factors influencing the PCD of Ti_*x*_Fe_2–*x*_O_3_ photoanode. This study confirms that RSM-based FC-CCD can be efficiently applied for the modeling and optimization of photocurrent response of Ti_*x*_Fe_2–*x*_O_3_ photoanodes.

## 1 Introduction

Photoelectrocatalysis (PEC) is considered one of the most promising approaches for harnessing solar energy to drive redox processes, including water splitting, oxidation of persistent organic pollutants,^1−3^ reduction of CO_2_^4,5^ and toxic inorganic ions,^6,7^ and inactivation of pathogenic microbes.^8,9^ In photoelectrocatalysis, a semiconductor material is used as the photoelectrode to absorb solar radiation and generate charge carriers (e^–^ and h^+^). The application of bias potential improves the separation of the photogenerated charge carriers to participate in redox reactions on the photoelectrode surface.^10^ Several semiconductor materials have been explored since Fujishima and Honda’s original demonstration of PEC water splitting with a TiO_2_ photoelectrode. Among the evaluated candidates, hematite (α-Fe_2_O_3_) stands out due to its suitable band gap energy (1.9–2.2 eV) for visible light absorption, high chemical stability in the electrolyte solution of pH 4–14, nontoxicity, abundance, and low cost.^11−13^ When subjected to one-sun illumination, α-Fe_2_O_3_ with a band gap energy of 2.2 eV may yield a maximum photocurrent density of 12.6 mA/cm^2^ at 1.23 V vs RHE, which corresponds to a solar-to-hydrogen (STH) efficiency of 16.8%.^12^

Achieving the theoretical maximum photocurrent density of α-Fe_2_O_3_ is challenging, however, due to the high recombination rate of photoexcited carriers in both the bulk material and at the α-Fe_2_O_3_/electrolyte interface. The elevated recombination rate is primarily attributed to the extremely short minority carrier (h^+^) diffusion length (2–4 nm) in α-Fe_2_O_3_.^12,14,15^ Therefore, nanostructuring or decreasing the film thickness can mitigate bulk carrier recombination as well as enhance charge separation by increasing the relative proportion of the space charge region within the total film thickness. Nevertheless, a thin α-Fe_2_O_3_ film may face intrinsic limitations in light absorption due to its small thickness in comparison to the large light penetration depth (∼120 nm).^16,17^ Due to this significant disparity between the light penetration depth and minority carrier diffusion length in α-Fe_2_O_3_, improving one factor generally entails compromises in the other. To solve this problem, several strategies have been adopted, for example, the development of nanostructured morphology, heteroatom doping, and surface engineering.^12,18−20^ Among these approaches, heteroatom doping is considered a promising technique to break down the compromise between light absorption and carrier transport.^11^ Incorporation of aliovalent dopants such as Ti^4+^,^21^ Sn^4+^,^22^ Si^4+^,^23^ and Pt^4+^^24^ has been found to improve the performance of hematite photoelectrodes by enhancing the mobility and/or longevity of minority carriers, thereby allowing a greater number of minority carriers to diffuse to the semiconductor/electrolyte interface.

The photocatalytic activity and optoelectronic properties of both undoped and doped hematite thin films are significantly affected by the synthesis conditions, as well as the concentration and distribution of dopant elements. Consequently, a variety of techniques encompassing electrodeposition,^25,26^ magnetron sputtering,^27,28^ hydrothermal method,^29,30^ chemical vapor deposition (CVD),^31,32^ spray pyrolysis^33,34^ and atomic layer deposition (ALD)^35,36^ have been extensively investigated for the production of hematite thin films. In comparison to the other deposition methods, ALD offers superior control over film thickness, elemental composition, uniformity, and conformality across extensive surface areas and complex 3D structures.^37−39^ In ALD, a surface is exposed to a metal precursor and a coreactant in alternating cycles, and the film growth propagates via self-limiting reactions on the surface. The cyclic pulsing characteristic of ALD ensures subangstrom-level precision in film thickness control. Furthermore, the dopant concentration of the thin films can be easily adjusted by varying the ALD cycle ratio of the host and dopant materials. In this study, we employed ALD to fabricate thin films of hematite and Ti-doped hematite.

To find the optimum deposition conditions, a response surface methodology (RSM) based experimental design was used. The traditional ‘one-variable-at-a-time’ optimization method entails a series of sequential experiments that are both expensive and time-consuming, yet it inadequately reveals interaction effects between the variables involved. In contrast, RSM is a robust technique that enables the design of experiments aimed at evaluating both the independent and interactive impacts of different experimental variables, while simultaneously minimizing the number of experiments required.^40,41^ Moreover, the RSM methodology also enables the development of a model and optimization of the desired responses. As a result, RSM-based experimental designs have been widely used in recent years for optimization purposes in various fields, including catalysis,^42,43^ energy conversion and storage^44,45^ and water treatment processes.^46,47^ The optimization of ALD processes using RSM-based experimental designs has also been investigated for various applications.^48,49^

In this study, we applied face-centered central composite design (FC-CCD), which is a simplified version of Central Composite Design (CCD), to optimize the photocurrent response of atomic layer deposited Ti-doped hematite (Ti_*x*_Fe_2–*x*_O_3_) photoanode. The film thickness (depends on the number of ALD cycles), dopant concentrations (depends on the TiO_2_ to Fe_2_O_3_ cycle ratio), and deposition temperature were chosen as the independent factors, and the photocurrent density was taken as the response of the model. To the best of our knowledge, RSM-based optimization of photocurrent response has not been previously applied for the hematite photoanode deposition using ALD.

## 2 Experimental Section

### 2.1 Fabrication of the Fe_2_O_3_ and Ti_*x*_Fe_2–*x*_O_3_ Photoelectrode

Thin films of Fe_2_O_3_ and Ti_*x*_Fe_2–*x*_O_3_ were deposited on conductive fluorine-doped tin dioxide (FTO)-coated glass (6–8 Ω/sq) and reference Si(100) substrates in a commercial flow-type hot-wall F-120 reactor (ASM Microchemistry). Prior to deposition, the FTO substrates were cleaned in an ultrasonic bath with industrial strength Branson solution, deionized water, ethanol and deionized water in a sequence of 10 min each. After cleaning, the substrates were dried with N_2_ blowing and stored in a dust-free environment.

The Fe precursor utilized for the deposition of Fe_2_O_3_ was ferrocene (FeCp_2_, 98%, obtained from Sigma-Aldrich), and the counter reactant was moist ozone (O_3_ + H_2_O). Previous studies on this process demonstrated that moist ozone yields more uniform Fe_2_O_3_ films than dry ozone (O_3_).^50^ For the deposition of Fe_2_O_3_ film, FeCp_2_ was sublimed at 65 °C from a semiopen glass boat inside the reactor and pulsed onto the substrate via inert gas valving. Ozone was generated at a concentration of 100 g/Nm^3^ using an ozone generator (Wedeco Ozomatic) from 99.999% O_2_ (WOIKOSKI) and pulsed into the reactor simultaneously with water vapor via solenoid and needle valves. The optimized cycle times of the Fe_2_O_3_ process were 3 s FeCp_2_ pulse/2 s purge/4 s O_3_ + H_2_O pulse/3 s purge (Figure S1).

**Figure 1:**
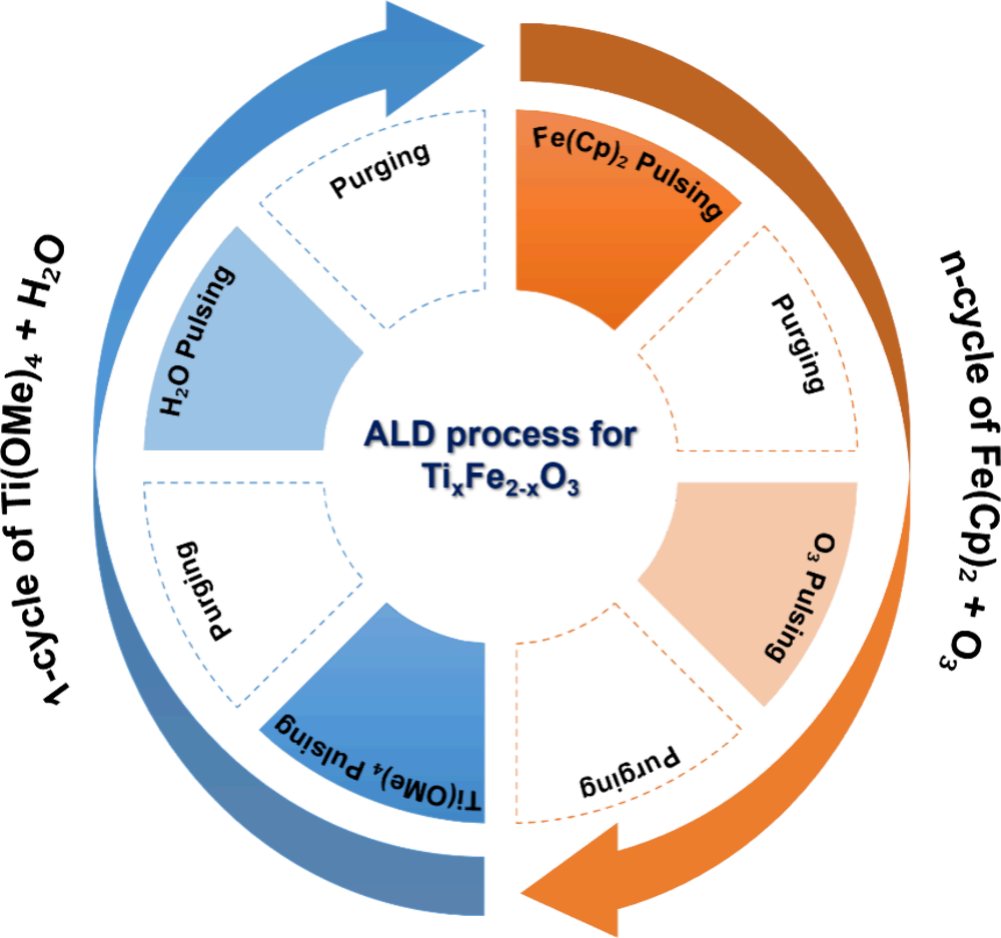
Schematic of the ALD supercycle sequences used for the deposition of Ti_*x*_Fe_2–*x*_O_3_.

For TiO_2_ deposition, titanium(IV) methoxide (Ti(OMe)_4_, 99.99%, Sigma-Aldrich) and water with cycle times of 0.6 s Ti(OMe)_4_ pulse/0.8 s purge/0.6 s H_2_O pulse/1 s purge (GPC = 0.43 Å) were used. Optimized deposition parameters were obtained from the work of Pore et al. using the similar ALD reactor.^51^ To introduce Ti-dopant into the Fe_2_O_3_ film, one FeCp_2_ + O_3_ cycle was replaced with one Ti(OMe)_4_ + H_2_O cycle inside a supercycle (as shown in Figure 1). By changing the ratio between the TiO_2_ and Fe_2_O_3_ cycles, the relative atomic concentration of Ti could be modified. When depositing the doped films, O_3_ was used with FeCp_2_ instead of moist ozone as it was found that uniform films with a relatively higher growth rate were obtained with only O_3_ pulsing.

### 2.2 Experimental Design for Photocurrent Optimization

**Figure 2:**
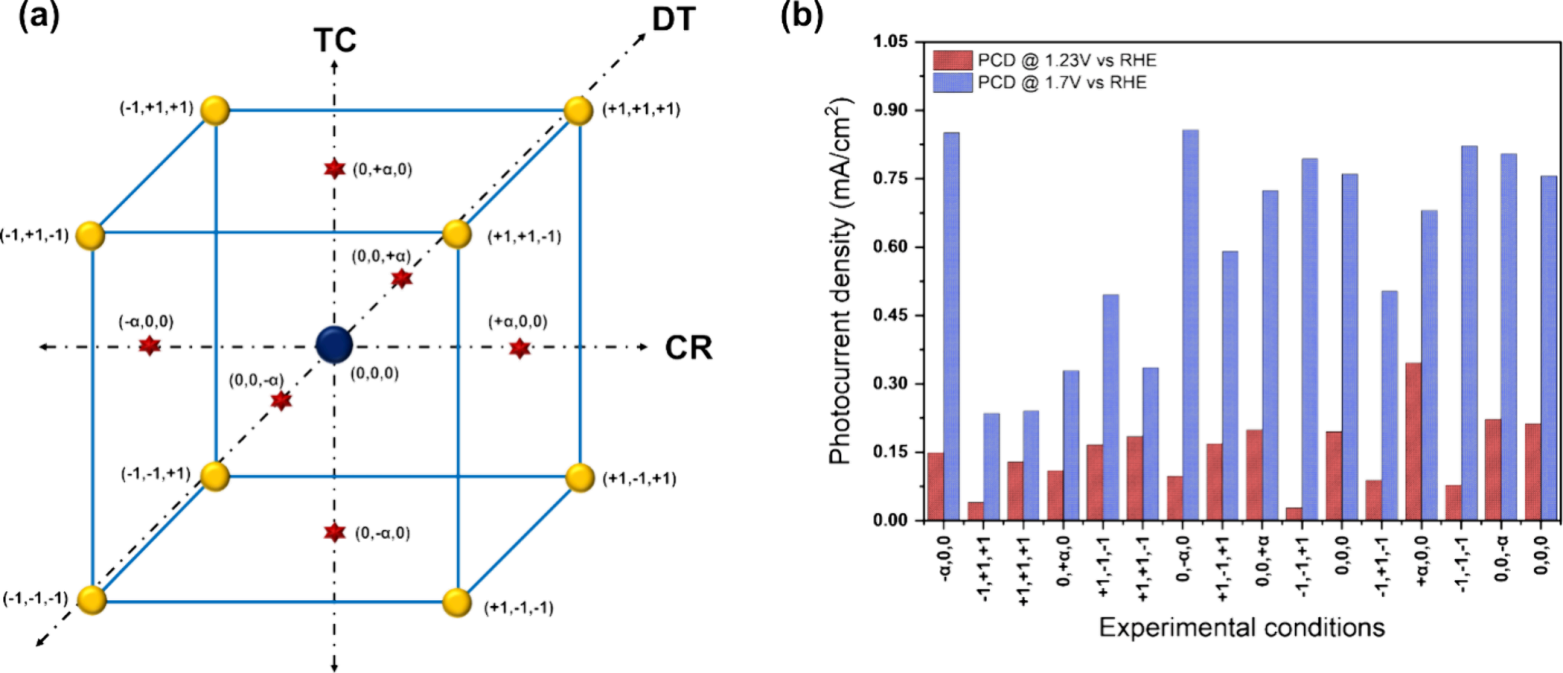
Graphical representation of experimental design points (a) and corresponding responses (b).

For photocurrent optimization, deposition experiments were designed according to Face Centered Central Composite Design (FC-CCD), which is a simplified version of Central Composite Design (CCD). The number of experiments in a CCD configuration comprises two-level factorial points (2^*k*^) together with axial points (2^*k*^) and center point (*C*_o_), as shown by eq 1.
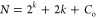
1where *k* symbolizes the number of independent variables (factors) and *N* represents the total number of experiments. For a system with three variables, CCD consists of 2^3^ = 8 factorial points, 2 × 3 = 6 axial points, and 2–5 center point depositions. The center point depositions were utilized for the computation of experimental error. The placement of the axial points in the experimental domain is specified by α = [2^*k*^]^1/4^, where α is the distance between the axial and center points. Depending on the α-value, the design exhibits several geometric arrangements, including spherical, orthogonal, or face-centered (FC). Owing to the experimental limitations on the levels of the independent variables, we employed an FC-CCD, in which α = ±1 and situated at the center of each face of the design space as presented in Figure 2a. The relationship between the coded levels (*x*_*i*_) and the actual deposition parameters (*X*_*i*_) used in the experimental design is defined by eq 2.
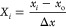
2where *x*_o_ and Δ*x* denote the actual value of *X*_*i*_ at the center point and the step change of *X*_*i*_ in actual form, respectively. Table 1 displays the independent deposition parameters together with their respective coded values.

**Table 1:** Experimental Variables and Their Levels Used in FC-CCD

independent variables	symbol	lower level (−1)	central point (0)	higher level (+1)
cycle ratio (TiO_2_:Fe_2_O_3_) (*X*_1_)	CR	1/34	1/19	1/4
total number of cycles (*X*_2_)	TC	140	560	980
deposition temperature, °C (*X*_3_)	DT	250	275	300

The depositions were conducted in a randomized manner according to the experimental matrix (Table S1) generated by *JMP Pro 14.0* software. Following the deposition process, all the films were subjected to annealing at 500 °C for 1 h in air and evaluated for the photocurrent response using a photoelectrochemical cell. Photocurrent density of the films, evaluated at two distinct potentials: 1.23 V (water oxidation potential) and 1.70 V vs RHE, was considered as the desired response of the model.

The correlation between the responses and independent variables is determined by fitting them into the following second-order polynomial eq 3:
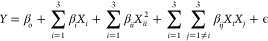
3Here, *Y* represents the response of the model, *k* indicates the total number of independent variables, β_o_ corresponds to the intercept of the model equation, while the coefficients β_*i*_, β_*ii*_*,* and β_*ij*_ reflect the values associated with linear, quadratic, and interaction effects, respectively. *X* denotes the independent variables and ε is a random error.

### 2.3 Photoelectrochemical Measurements

Photoelectrochemical measurements were carried out in a standard three-electrode setup using the fabricated photoanode as the working electrode, a commercial platinum mesh as the counter electrode, and an Ag/AgCl electrode as the reference electrode. The active area of the working electrode was 0.24 cm^2^. The photoelectrode was irradiated from the back side using a solar simulator (SciSun-300, class AAA) equipped with an AM 1.5G filter at an adjusted intensity of 100 mW/cm^2^. The photocurrent density was detected in an aqueous solution containing 0.1 M NaOH (pH 13.2) by linear sweep voltammetry (LSV) using an Autolab PGSTAT302N potentiostat/galvanostat. The scan was performed in both dark and simulated light conditions at a scan rate of 50 mV/s. All the measured potentials were converted to the potential vs reversible hydrogen electrode (RHE) using the following Nernst equation (eq 4):
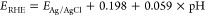
4Here, *E*_Ag/AgCl_ is the measured potential vs the Ag/AgCl reference electrode. Electrochemical Impedance Spectroscopy (EIS) analysis was carried out using the same 3-electrode setup and 0.1 M NaOH electrolyte from 100 kHz to 0.2 Hz at 1.23 V vs RHE potential under one sun illumination. The data were recorded using a PalmSens EmStat4s potentiostat and fitted with the equivalent circuit model using PSTrace software. Mott–Schottky plots were recorded in the dark at a frequency of 1.0 kHz with an AC amplitude of 10 mV, and electrodes were scanned from 0.4 to −0.9 V against the Ag/AgCl reference electrode.

### 2.4 Photoelectrode Characterization

The surface morphology of the Fe_2_O_3_ and Ti_*x*_Fe_2–*x*_O_3_ was observed with a Hitachi S-4800 field-emission scanning electron microscope (FESEM). Energy dispersive X-ray spectroscopy (EDS) measurements were done with an Oxford INCA 350 energy spectrometer connected to the FESEM. k-ratios of FeKα and TiKα obtained from the EDS measurements, as well as bulk density of TiO_2_ (4.23 g/cm^3^) and Fe_2_O_3_ (5.25 g/cm^3^), were used to calculate film thickness with the GMRFilm software.^52^ Crystallinity of the films was assessed with grazing incidence X-ray diffraction (GIXRD, incident angle 1°) with a Rigaku Smartlab diffractometer utilizing Cu Kα-radiation. Raman spectra were obtained using an NT-MDT Ntegra spectrometer in a confocal geometry with a 100× objective and a red laser (633 nm). The incident laser power was 9.24 mW, and each sample was measured with 10 exposures lasting 30 s each. X-ray photoelectron spectroscopic (XPS) analysis was performed in ultrahigh vacuum (10^–10^ mbar) using a PREVAC system equipped with an EA-15 hemispherical electrostatic energy analyzer and an RMC50 monochromatic X-ray source featuring an Al Kα anode (1486.7 eV). The analyzer pass energy was 200 and 100 eV for the survey scan and high-resolution individual elemental scan, respectively. XPS spectra were analyzed using Casa XPS software.

## 3 Result and Discussion

### 3.1 Atomic Layer Deposition of Fe_2_O_3_ and Ti_*x*_Fe_2–*x*_O_3_

Before conducting depositions using the experimental design, ALD processing parameters such as surface saturation studies and growth per cycle (GPC) estimation were carried out. Additionally, preliminary characterization of the photoelectrodes was performed to verify the presence of the desired phase in the deposited film. To achieve surface saturation of Fe_2_O_3_, the ALD cycle time was optimized on a Si substrate at 300 °C, and the results are presented in Figure S1a,b. It can be seen that the saturated growth was obtained at 3 s pulse time for FeCP_2_, whereas for the O_3_ pulsing, it was 3 s. However, to improve film uniformity 4 s pulse time was adopted for the rest of the study.

The GPC of Fe_2_O_3_ and TiO_2_ on Si(100) substrates was measured to be 0.74 and 0.43 Å, respectively, at 300 °C (Figure S1c). When a single cycle of TiO_2_ was introduced after every four cycles of Fe_2_O_3_, the GPC increased to 0.9 Å (Figure S1d), accompanied by improved film uniformity. This enhancement in the GPC and uniformity is likely due to the surface passivation effect of the TiO_2_ layer, which prevents Fe-precursor or O_3_ decomposition caused by the catalytic effect of the Fe_2_O_3_ surface. A similar growth behavior has also been reported by Li et al.^53^

**Figure 3:**
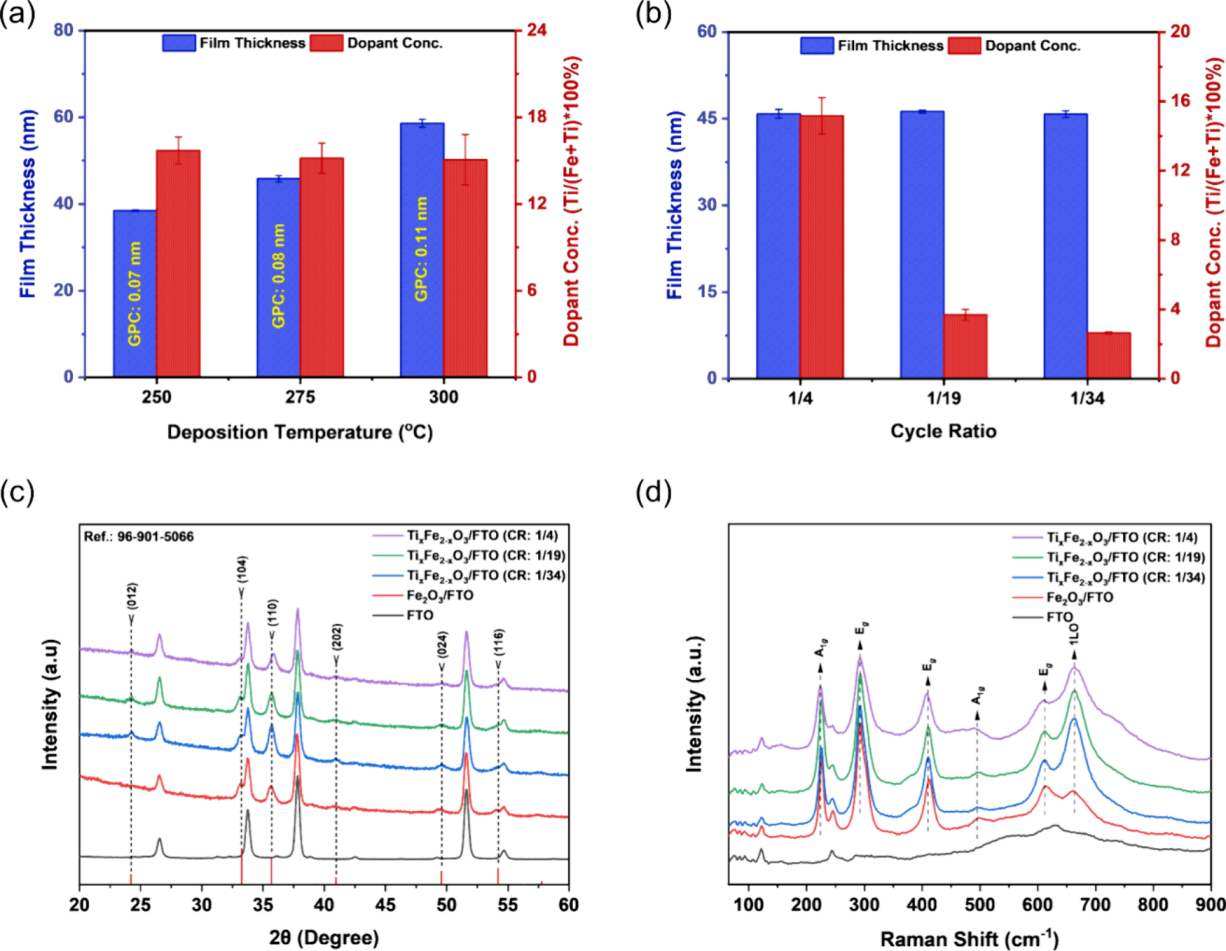
Film thickness and dopant concentration as a function of (a) deposition temperature (cycle ratio: 1/4) (b) Ti to Fe cycle ratio (deposition temperature: 300 °C), (c) XRD patterns and (d) Raman spectra of Fe_2_O_3_ and Ti_*x*_Fe_2–*x*_O_3_ samples. All the films were annealed at 500 °C for 1.0 h after deposition.

Figure 3a represents the film thickness and dopant concentration of Ti_*x*_Fe_2–*x*_O_3_ thin films as a function of the deposition temperature. All the films were deposited using 560 cycles with a TiO_2_ to Fe_2_O_3_ cycle ratio of 1:4. It can be seen that the GPC increased from 0.7 to 1.1 Å with the increasing deposition temperature from 250 to 300 °C, while the dopant concentration was slightly decreased, from 15.7 ± 0.9 to 15.1 ± 1.8 at %.

Film thickness and dopant concentration as a function of the TiO_2_ to Fe_2_O_3_ cycle ratio are presented in Figure 3b. All the films were deposited using 560 ALD cycles while the cycle ratio was varied from 1:4 to 1:34. Atomic concentrations of Ti were obtained as 15.2 ± 1.1, 3.7 ± 0.3, and 2.6 ± 0.1% with the TiO_2_ to Fe_2_O_3_ cycle ratios of 1:4, 1:19, and 1:34, respectively. No significant change in film thickness was observed with the different cycle ratios.

The GIXRD patterns of both Fe_2_O_3_ and Ti_*x*_Fe_2–*x*_O_3_ films deposited on the FTO substrates are presented in Figure 3c. The X-ray diffractograms are primarily dominated by peaks corresponding to the underlying FTO substrate, with additional well-defined peaks indicating the presence of the hematite (α-Fe_2_O_3_) phase. Initially, the doped films exhibited an amorphous structure, but after annealing in air at 500 °C, a crystalline hematite phase appeared with no obvious changes in crystal structure. All the Ti_*x*_Fe_2–*x*_O_3_ films were grown using 980 cycles, while the Fe_2_O_3_ film was grown using 1000 cycles.

Raman spectra of the Fe_2_O_3_ and Ti_*x*_Fe_2–*x*_O_3_ thin films are presented in Figure 3d. Both Fe_2_O_3_ and Ti_*x*_Fe_2–*x*_O_3_ samples exhibit six vibrational bands at 226, 293, 411, 498, 614, and 662 cm^–1^. According to the Group theory prediction, the peaks at 226 and 498 cm^–1^ belong to the A_1g_ mode, whereas the peaks at 293, 411, and 614 cm^–1^ correspond to the E_g_ mode. The appearance of the Raman forbidden longitudinal optical (LO) mode at 662 cm^–1^ indicates structural disorder-induced symmetry breakdown for the scattering LO phonon. The doped films exhibited a significant rise and broadening of the LO peak in comparison to the binary Fe_2_O_3_ film. The intensity of the peak has a positive correlation with the concentration of Ti in the doped films.

**Figure 4:**
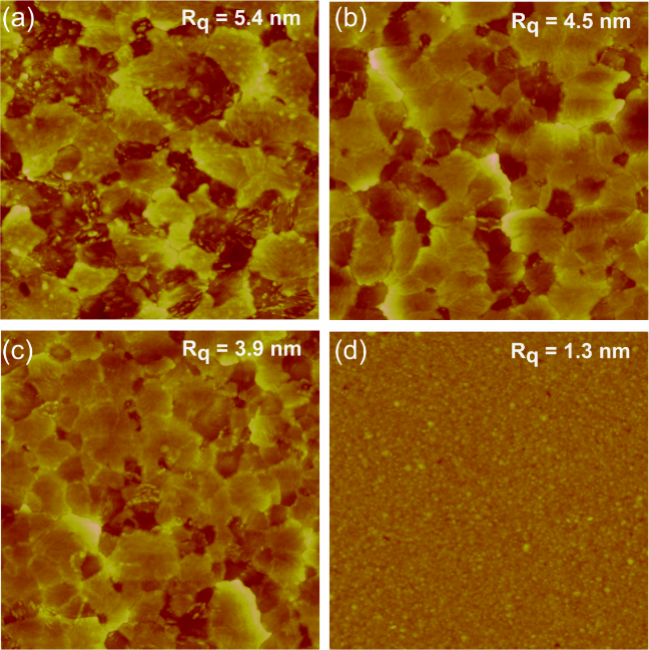
Surface morphology of Fe_2_O_3_ and Ti_*x*_Fe_2–*x*_O_3_. AFM images (2 × 2 μm^2^) on the Si substrate after annealing at 500 °C in air (a) Fe_2_O_3_ (b–d) Ti_*x*_Fe_2–*x*_O_3_ with CR 1/34, 1/19, and 1/4, respectively.

Surface morphologies and roughnesses of both Fe_2_O_3_ and Ti_*x*_Fe_2–*x*_O_3_ films were analyzed using AFM. Figure 4a–d shows the topographic surface images of the films grown on Si substrate using 560 ALD cycles at 300 °C. The Fe_2_O_3_ thin film exhibits the highest surface roughness of 5.4 nm. With Ti doping, the surface roughness decreases, and the smoothest film with a surface roughness of 1.3 nm was achieved when the TiO_2_ to Fe_2_O_3_ cycle ratio was 1:4.

### 3.2 Optimization of Photocurrent Response Using FC-CCD

For the optimization of the photocurrent response of Ti_*x*_Fe_2–*x*_O_3_ photoelectrodes, 16 different films were fabricated using ALD in accordance with the FC-CCD matrix (Figure 2a) in a randomized manner. Subsequently, these photoelectrodes were subjected to annealing at 500 °C for 1 h in air. Following the annealing, the samples were allowed to cool down to room temperature and their PCD was measured by LSV using a photoelectrochemical cell under one-sun illumination. The PCD data obtained at 1.23 and 1.70 V vs RHE are presented in Table S1, and their graphical representation is shown in Figure 2b. It is worth noting that the PCD obtained at 1.23 V varies between 0.03 and 0.35 mA/cm^2^. The lowest value of the PCD was achieved for the deposition condition of CR: 1/34, TC: 140, DT: 300 °C, while the highest value was obtained for the CR: 1/4, TC: 560, DT: 275 °C. Similarly, at 1.70 V, the lowest PCD value was found to be 0.23 mA/cm^2^ obtained with CR: 1/34, TC: 980, DT: 300 °C, while the maximum value of 0.86 mA/cm^2^ was achieved with CR: 1/19, TC: 140, DT: 275 °C.

The mathematical models obtained after fitting the second-order polynomial eq 3 to the experimental results are presented as eqs 5 and 6. These equations establish relations between the response variables and the independent variables at the coded level and contain all terms, regardless of their statistical significance.
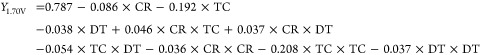
5
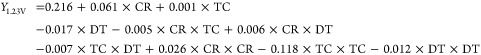
6

The coefficients were determined using the least-squares method, and the sign (+ or −) indicates the trend of the relationship between the factor and its response. The photocurrent response is positively impacted by the factors with positive coefficients and negatively impacted by those with negative coefficients. The absolute value of a coefficient is a measure of the strength of the correlation between the factor and the response.

**Figure 5:**
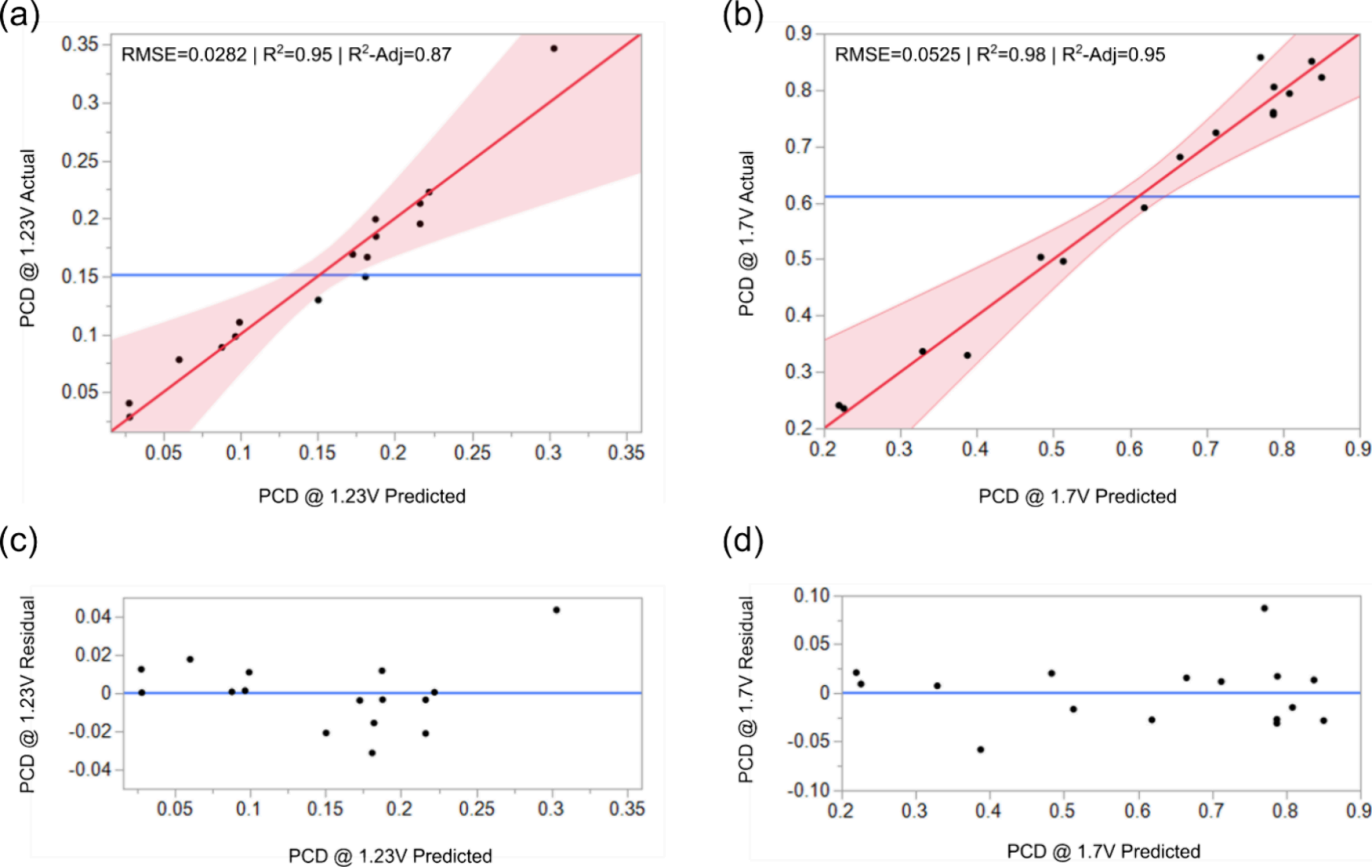
Graphical representation of actual vs predicted values of PCDs (a and b) and their corresponding residuals (c and d).

Figure 5 illustrates correlations between the experimental and predicted values of the photocurrent densities. All the experimental data points, except for one, fall inside the significance level curves and in close proximity to the predicted straight line. This indicates that the values projected with the models are highly accurate and closely aligned with the actual values. The coefficients of determination (*R*^2^) values, 0.95 and 0.98 for PCD at 1.23 and 1.70 V, respectively, are approaching unity and thereby suggesting that the regression model effectively establishes a strong relationship between the deposition parameters and the photocurrent response of the Ti_*x*_Fe_2–*x*_O_3_ photoanodes. Adjusted *R*^2^ (*R*^2^-Adj) also serves as an indicator of the adequacy of a regression model, accounting for both the number of experiments and the number of terms within the model by incorporating degrees of freedom into its calculations. When a model contains numerous terms relative to the number of experiments, the *R*^2^-Adj value may appear noticeably smaller compared to the *R*^2^ value. In this study, the *R*^2^-Adj values for PCD at 1.23 and 1.70 V were 0.87 and 0.95, respectively, closely aligned with the corresponding *R*^2^ values. Besides the correlation coefficient, the distribution of residuals around the mean is also a significant factor in assessing the adequacy of regression models. This distribution enables the assessment of unobservable experimental error. The absence of any obvious regular trends in the distribution of residuals around the projected line (Figure 5c,d) suggests the absence of any potential issues.

**Figure 6:**
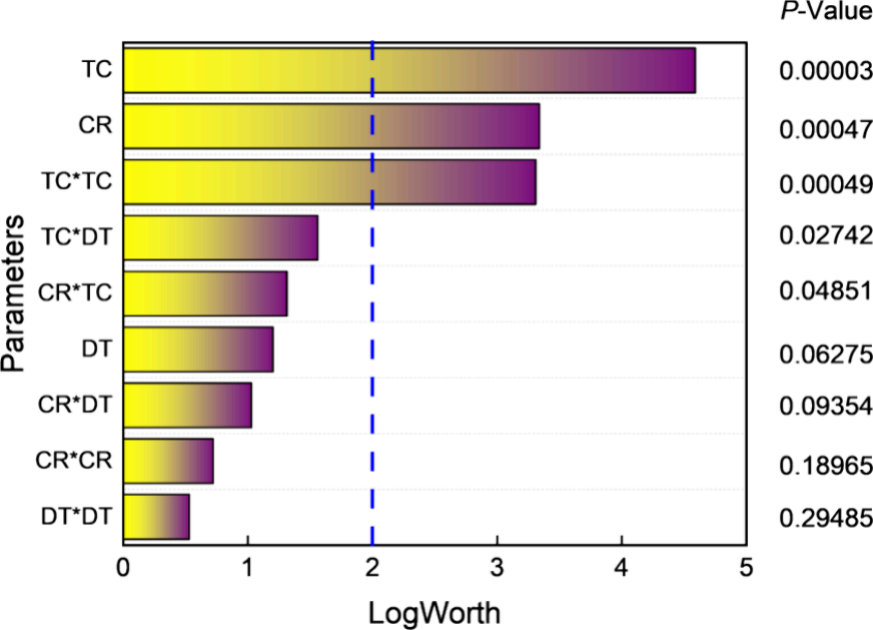
Overall effect summary.

The overall significance of the model parameters, based on the *P*-value and LogWorth, is presented in Figure 6. LogWorth is defined as −log10(*P*-value), which transforms *P*-values into a scale suitable for graphical representation. Generally, a *P*-value of less than 0.01 suggests a strong indication against the null hypothesis, i.e., the operating variable has no correlation with the response. The reference blue line represents −log10(0.01), which equates to a LogWorth of 2.0, establishing a threshold for significance. Thus, any parameter with a LogWorth exceeding 2.0 is considered significant. Given these criteria, the total number of cycles (TC) has the most substantial impact on the photocurrent density with a LogWorth of 4.59, followed by the cycle ratio (CR) and the quadratic interaction of TC (TC*TC) with LogWorth values of 3.32 and 3.33, respectively. The other parameters appear to be less significant. Based on this analysis, it can be concluded that the photocurrent density is primarily influenced by TC, CR, and TC*TC, and the model can sufficiently predict the photocurrent density. The significant quadratic effect of TC suggests that the optimal thickness level lies within the experimental range, rather than at the extreme boundaries.

Table 2 represents the outcomes of the analysis of variance (ANOVA) conducted to assess the fitting of the quadratic model, essential for evaluating its significance and adequacy. ANOVA evaluates the variability attributed to the model against that arising from experimental error, thereby determining whether the variation of the model is statistically significant relative to the residual error. This evaluation relies on the *F-*value, computed as the ratio of the mean square error of the model to the residual. A satisfactory model should yield an *F*-value surpassing the tabulated *F*-value for a given number of degrees of freedom at a significance level σ. In this study, the model *F*-values were computed as 12.38 and 27.33 for the resulting photocurrent density at 1.23 and 1.7 V, respectively. These values notably exceed the tabulated *F*-value (*F*_9,6_ = 4.1) for a significance level (σ) of 0.05, suggesting that these models effectively predict experimental results with a high level of confidence.

**Table 2:** ANOVA for the Response Surface Model

	DF		sum of squares		mean square		*F* ratio		*P*-value	
source	1.23 V	1.70 V	1.23 V	1.70 V	1.23 V	1.70 V	1.23 V	1.70 V	1.23 V	1.70 V
model	9	9	0.089	0.737	0.0099	0.082	12.38	27.33	0.0030	0.0003
error	6	6	0.005	0.017	0.0008	0.003				
C. total	15	15	0.094	0.754						

**Figure 7:**
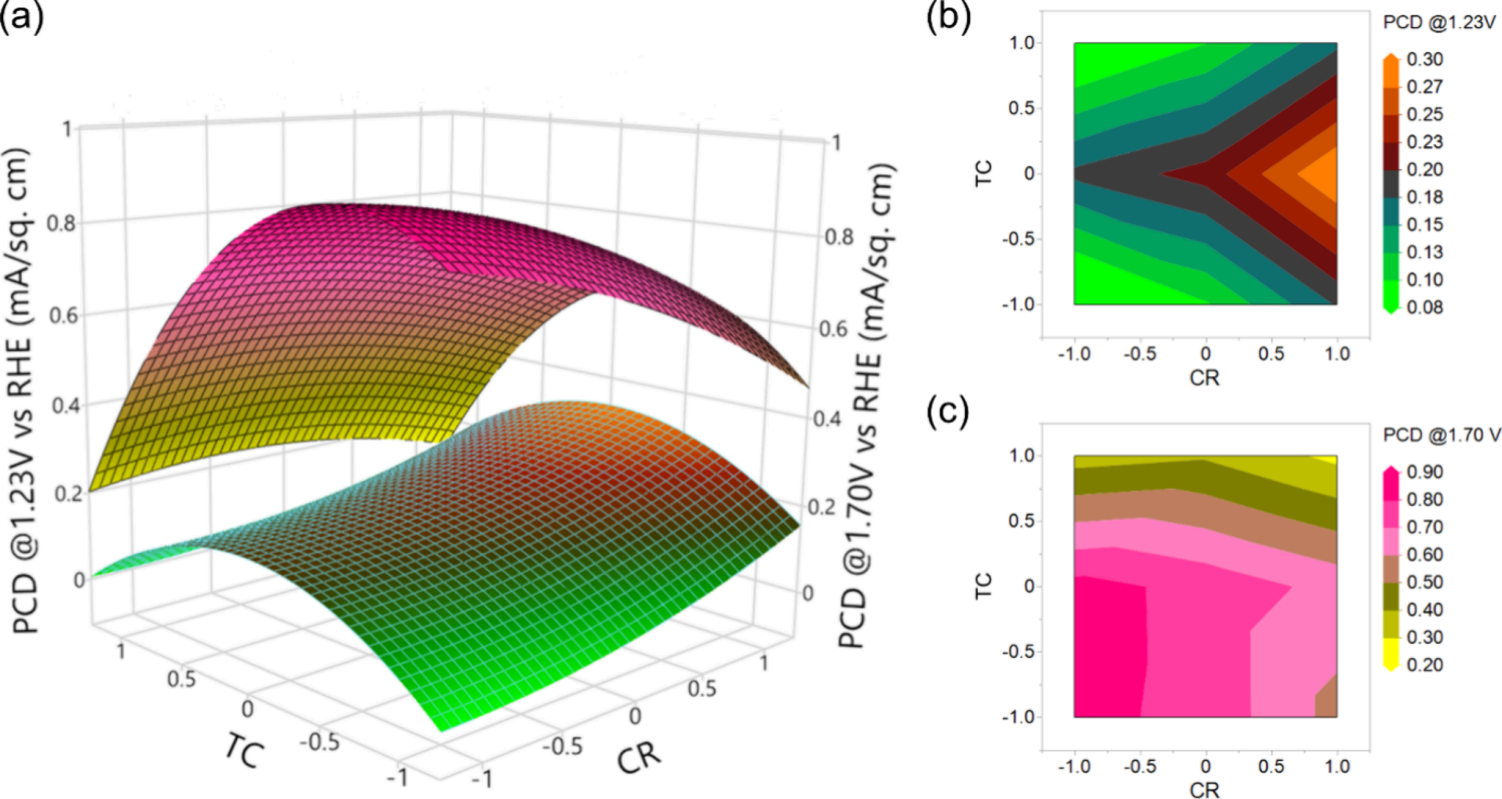
Response surface (a) and contour plots (b and c) of PCD as a function of TC and CR interactions.

The regression model equation can also be visually depicted through 3D response surface plots and 2D contour plots, enabling a comprehensive understanding of the interactive relationships between the independent variables and the corresponding responses. Figure 7 illustrates the 3D response surface plot (a) and the corresponding contour plots (b, c) where the photocurrent densities of Ti_*x*_Fe_2–*x*_O_3_ photoanodes at 1.23 and 1.70 V were plotted as a function of two factors (TC and CR) at a time. The third factor, DT, is maintained at the center point condition, i.e., 275 °C. From Figure 7a, it is evident that the PCD at 1.23 V exhibits an upward trend with an increase in the CR, whereas PCD initially rises and then declines with an increasing number of TC. This interactive correlation is further elucidated in the 2D contour plot (Figure 7b), which illustrates that the maximum PCD at 1.23 V can be obtained at the highest design point of CR (1/4) and the central design point of TC (560 cycles). Conversely, the PCD at 1.70 V indicates an increasing trend with a decreasing value of both CR and TC. The maximum PCD at 1.70 V is observed to occur from the center to the lower design point. This observation is further supported by the contour plot (Figure 7c), which confirms that the peak PCD at 1.7 V can be obtained from the center point to the lower design point of both TC and CR.

**Figure 8:**
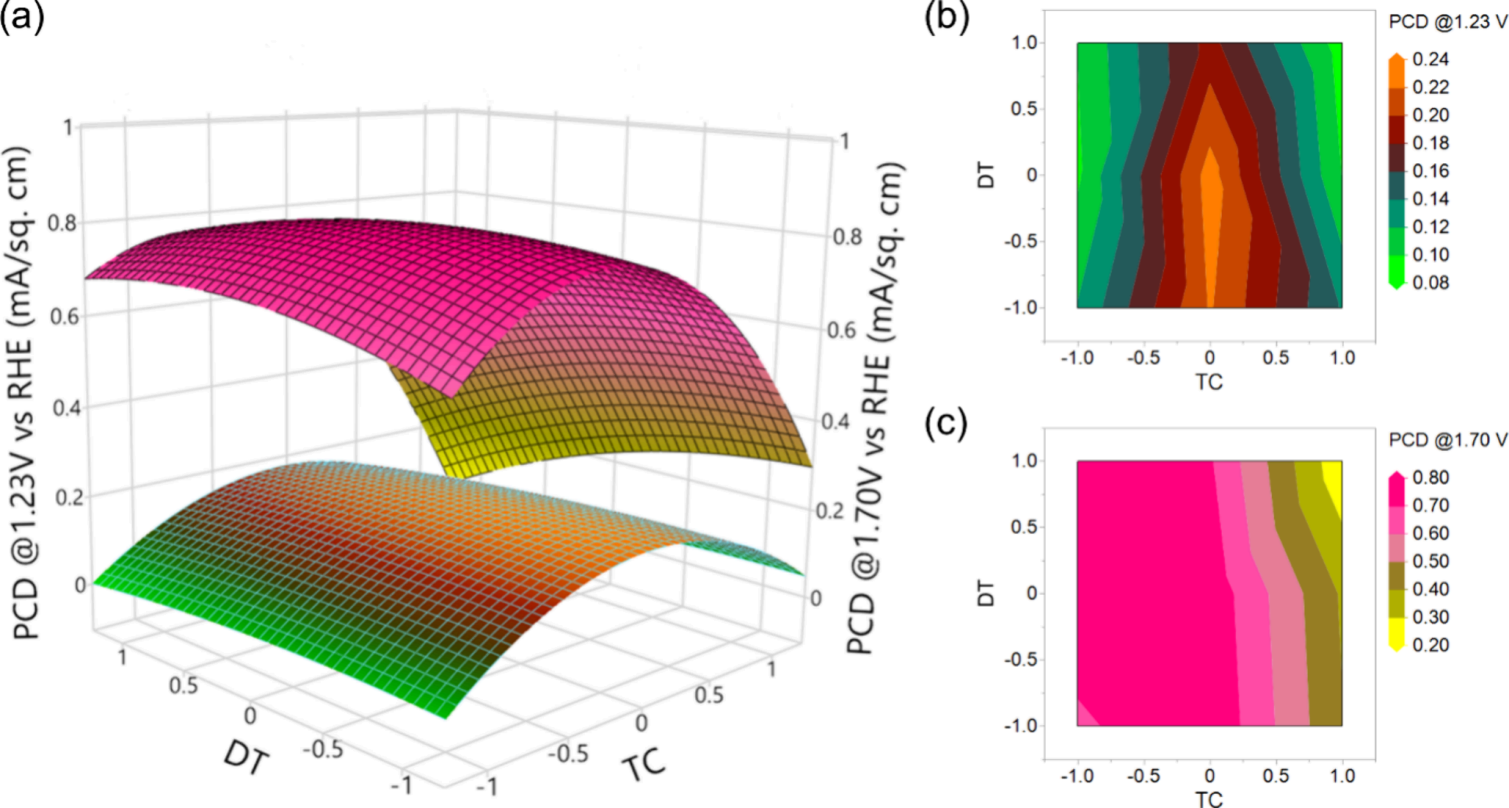
Response surface (a) and contour plots (b and c) of PCD as a function of DT and TC interaction.

Figure 8 illustrates the interactive effect of the TC and DT on the photocurrent densities of Ti_*x*_Fe_2–*x*_O_3_ photoanodes with the CR held constant at its center point (1/19). Notably, at the central design point of TC, a higher PCD at 1.23 V is achievable with relatively lower DT values, whereas for 1.7 V, the highest PCD occurs at the lower design point of TC and DT. Interestingly, DT appears to exert minimal influence on PCD at both 1.23 and 1.70 V, with a higher PCD region observed from the central to the lower design point at 1.23 V. The 2D contour plot further indicates that maximum PCD is achieved when TC is at its central design point (560 cycles), while temperature exhibits minimal impact on PCD (Figure 8b,c).

**Figure 9:**
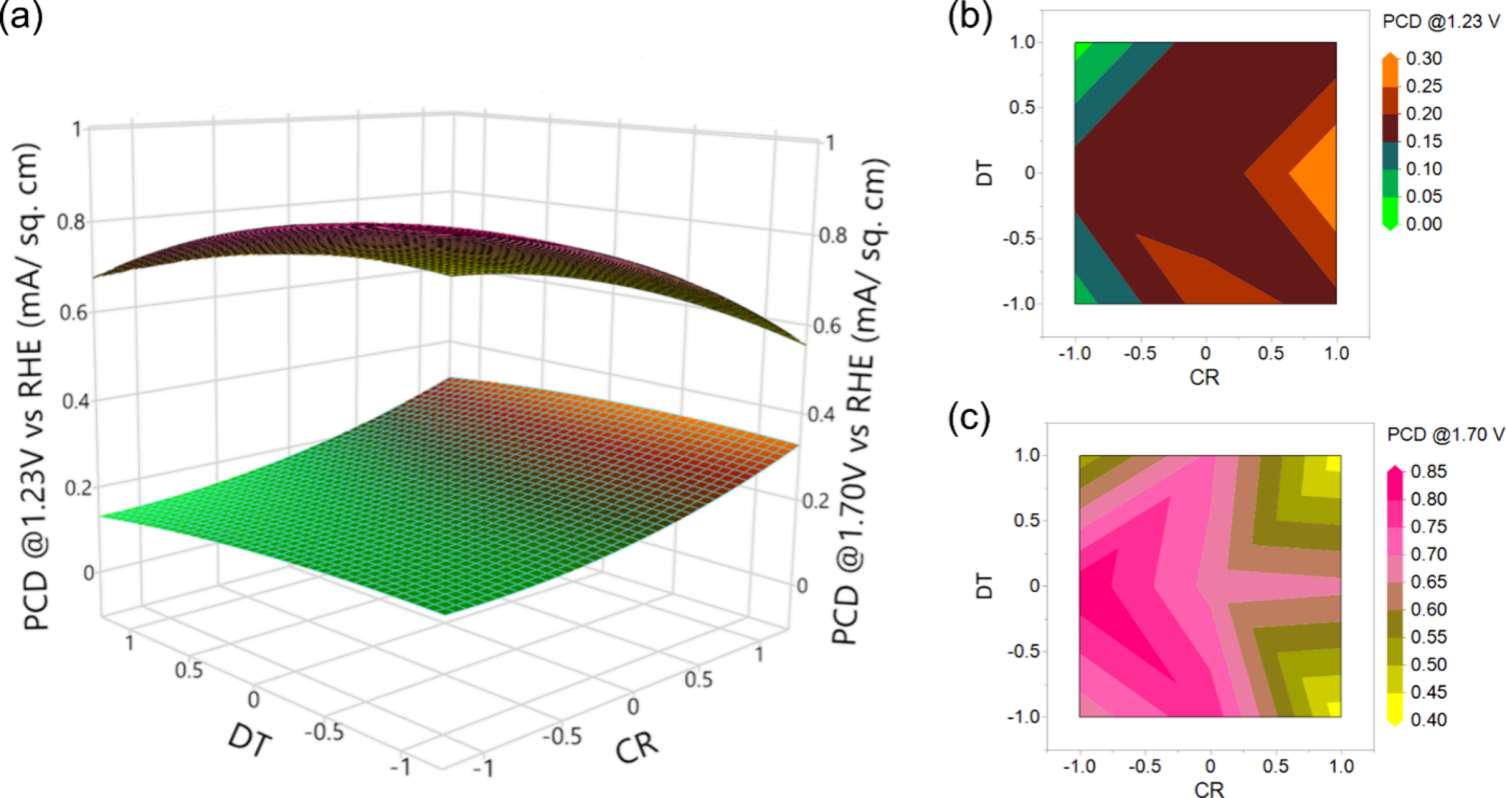
Response surface (a) and contour plots (b and c) of PCD as a function of DT and CR interactions.

The interactive correlation between the CR and DT on photocurrent densities of Ti_*x*_Fe_2__–*x*_O_3_ photoanodes is presented in Figure 9, while the third deposition parameter TC is held constant at its central design point (560 cycles). The response surface and contour plots reveal that minimal photocurrent response is observed at both high and low deposition temperatures. Notably, the maximum PCD is attained at the central design point DT (275 °C) for both applied potentials. Conversely, while the peak PCD at 1.23 V occurs at a higher design point of CR, at 1.70 V, it is observed at a lower design point.

### 3.3 Determination of Optimum Conditions and Validation of the FCCCD Model

**Figure 10:**
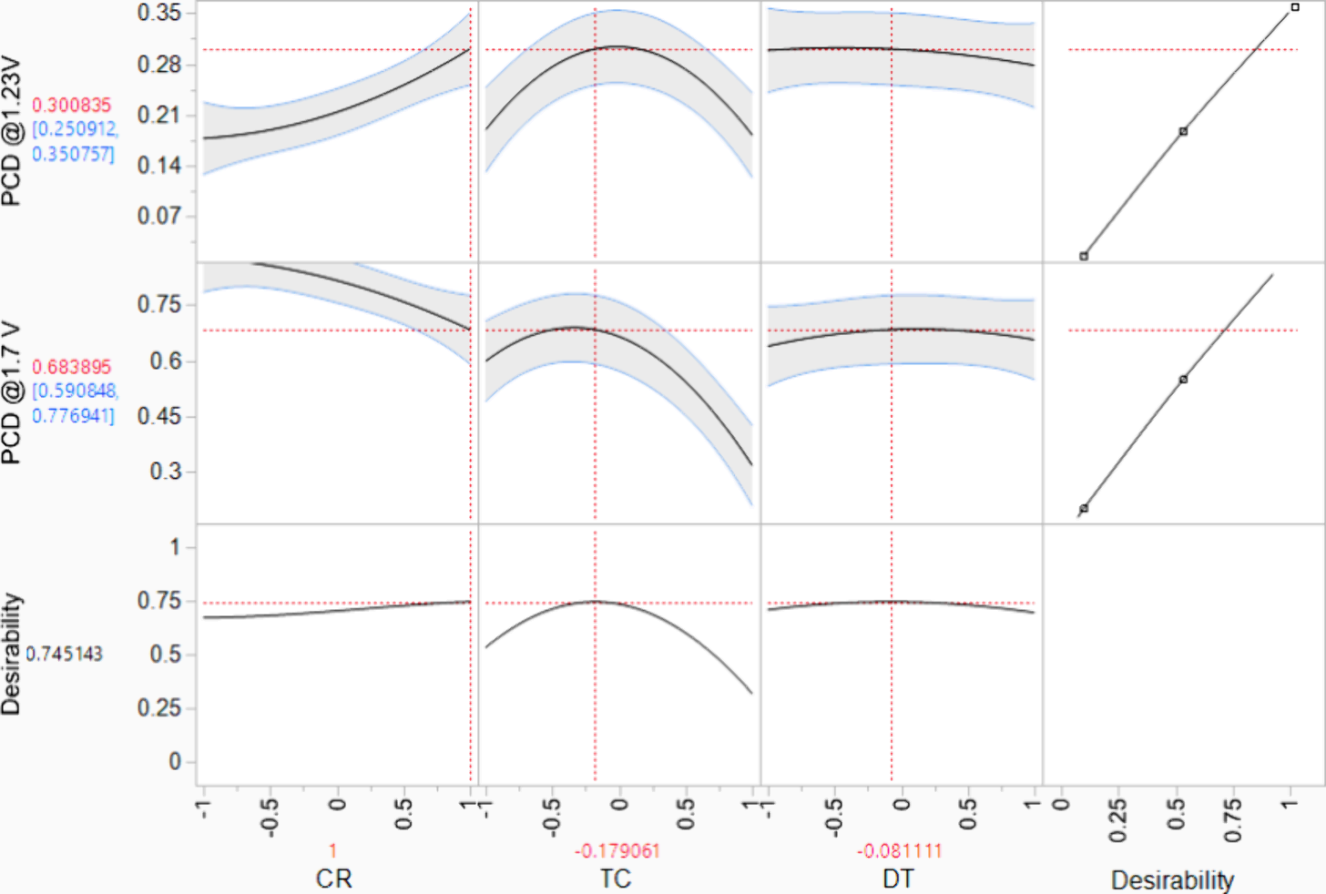
Desirability plot showing the precise deposition conditions of the three studied variables leading to the optimal PCDs. The shaded areas with the blue lines indicate the 95% confidence intervals. The dotted lines mark the points of maximum PCD.

The utilization of the RSM design in the optimization study allows systematic exploration of the experimental variables to attain an optimal response. The utilization of the desirability function, a robust optimization tool available within the JMP software, enabled execution of the optimization process. The predictive model derived from regression analysis effectively identified the optimal cycle ratio, total number of cycles, and deposition temperature. These parameters were found to yield a theoretical photocurrent density of 0.30 ± 0.05 mA/cm^2^ at 1.23 V and 0.68 ± 0.09 mA/cm^2^ at 1.70 V vs RHE, as depicted in Figure 10. These specified values were obtained with a 95% confidence level and correspond to a desirability rating of 74.51%.

**Figure 11:**
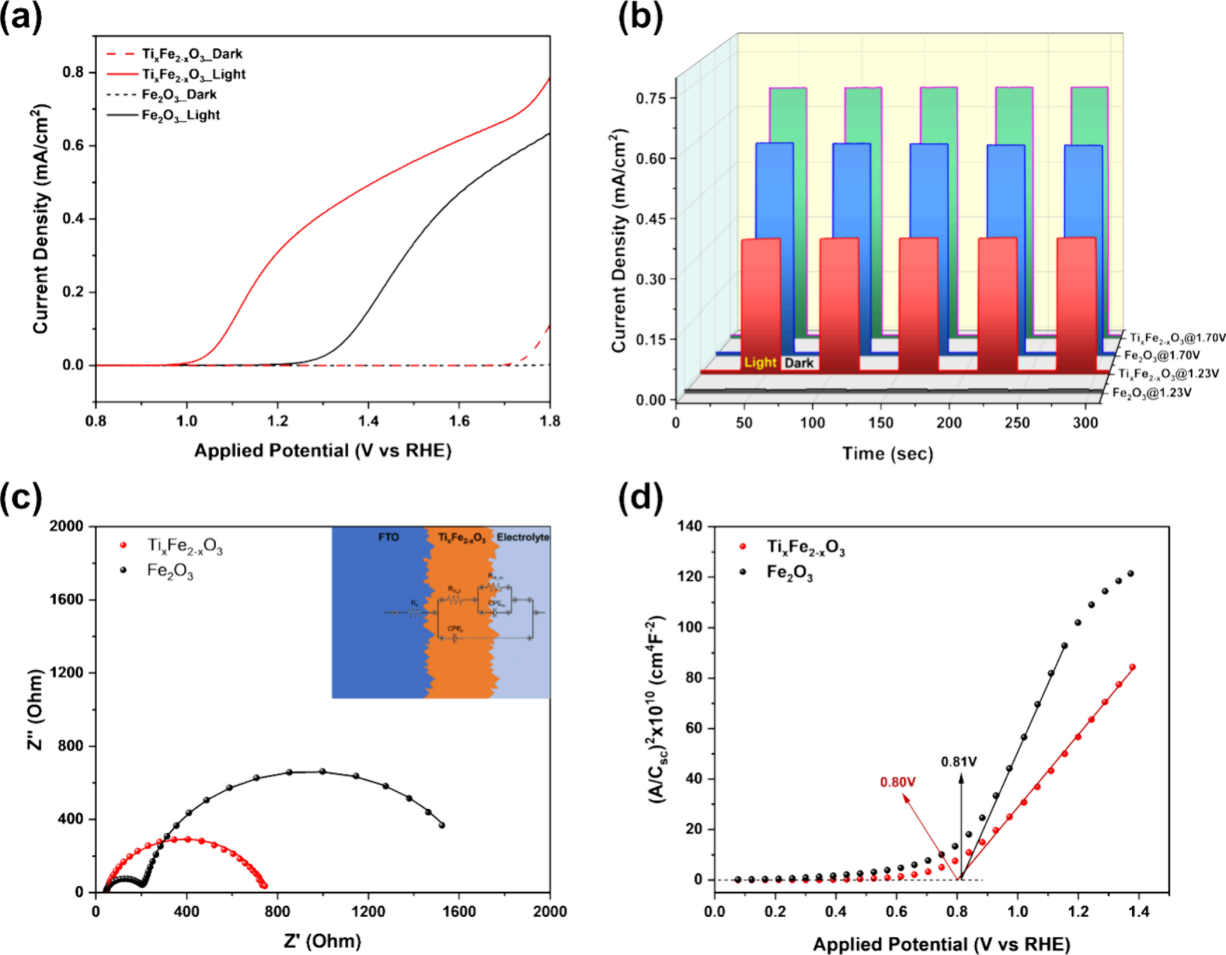
Photoelectrochemical characterization of the optimized Ti_*x*_Fe_2__–*x*_O_3_ and Fe_2_O_3_ photoanode (a) LSV analysis, (b) chronoamperometric analysis, (c) EIS, and (d) M–S plot.

To verify the modeling results, the coded values of the independent variables were converted into the actual values using eq 2, and a deposition was carried out according to the optimum deposition conditions outlined in Table 3. Following the deposition, the sample was annealed at 500 °C for 1 h in air. The film thickness and dopant concentration were obtained as 39.4 ± 0.2 nm (GPC: 0.08 nm) and 15.5 ± 0.7 ca %, respectively. PEC characterization of the optimized Ti_*x*_Fe_2__–*x*_O_3_ photoanode yielded photocurrent densities of 0.35 mA/cm^2^ at 1.23 V and 0.67 mA/cm^2^ at 1.70 V vs RHE (Figure 11a), both of which were within the error margins predicted by the model equations. Compared with the undoped hematite film deposited with the same number of ALD cycles, the doped one shows higher photocurrent density and lower onset potential. The corresponding chronoamperometric plots are presented in Figure 11b, which shows the light on–off cycling experiments conducted at 1.23 and 1.70 V vs RHE to compare the photocurrent with the dark current response.

**Table 3:** Optimum Deposition Conditions

parameters	coded value	decoded value	predicted PCD (mA/cm^2^)	experimental PCD (mA/cm^2^)
cycle ratio	+1	1/4	0.30 ± 0.05 @ 1.23 V	0.35 @ 1.23 V
total number of cycles	–0.18	∼490	0.68 ± 0.09 @1.70 V	0.67 @ 1.70 V
deposition temperature (°C)	–0.08	∼275		

EIS analysis was carried out to evaluate the effect of Ti doping on the charge transfer resistance in the bulk and surface of Fe_2_O_3_ and the optimized Ti_*x*_Fe_2__–*x*_O_3_ photoanode under 1-SUN illumination at an applied potential of 1.23 V vs RHE. The results are presented as Nyquist plots in Figure 11c. Two overlapping semicircles appeared for the sample without doping. The high frequency arc (1st semicircle) corresponds to the charge transport inside the bulk semiconductor, whereas the low frequency arc (2nd semicircle) is linked to the charge transfer at the semiconductor/electrolyte interface.^54^ The equivalent circuit model is presented in the top right corner, in which the parameters *R*_ct_b_, *R*_ct_ss_, and *R*_s_ correspond to the charge transfer resistance within the bulk, at the electrode/electrolyte interface, and series resistance associated with the electrolyte and FTO. By fitting the equivalent circuit model to the experimental data, it was observed that in the undoped sample, the charge transfer resistance at the electrode/electrolyte interface (1338 Ω) is significantly higher than the bulk resistance (167 Ω), indicating that interfacial processes predominantly limit charge transport. In contrast, the optimized Ti_*x*_Fe_2__–*x*_O_3_ sample exhibits a markedly lower interfacial charge transfer resistance (385 Ω). This result aligns well with previously published studies, which have demonstrated that Ti doping modifies surface state distributions, thereby enhancing charge transfer efficiency at the electrode/electrolyte interface.^50,54^

M-S analysis was conducted to determine the carrier concentration in both the doped and pristine photoelectrodes. As illustrated in Figure 11d, both photoelectrodes exhibit a positive slope, characteristic of n-type conductivity. By extrapolating the linear region of the plot to the potential axis, the flat-band potentials were determined to be 0.80 and 0.81 V vs RHE for the Ti_*x*_Fe_2__–*x*_O_3_ and Fe_2_O_3_ photoelectrodes, respectively. These values align well with the literature, which reports that Ti-doping results in a cathodic shift in the flat-band potential.^25,55^ Notably, the flat-band potentials of both the Fe_2_O_3_ and Ti_*x*_Fe_2__–_*x*O_3_ films are more cathodic than the photocurrent onset potentials (as shown in Figure 11a). This difference between the positive onset potential and the flat-band potential can be attributed to electron–hole recombination due to surface states or the slow kinetics of water oxidation.^55^ The charge carrier concentrations, derived from the slopes of M–S plots, were 1.8 × 10^18^ and 3.2 × 10^18^ cm^–3^ for Fe_2_O_3_ and Ti_*x*_Fe_2–*x*_O_3_ photoelectrode, respectively. This provides direct evidence that Ti doping enhances donor concentration due to the incorporation of Ti into the Fe_2_O_3_ lattice. The increased donor concentration decreases the width of the space charge layer, thereby intensifying the electric field within the depletion region. The depletion layer width (*w*) was calculated using eq 7:
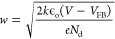
7where *k* is the dielectric constant (a value of 32 is considered for Fe_2_O_3_), ϵ_o_ is the vacuum permittivity (8.85 × 10^–14^ CV^–1^ cm^–1^), *V* is the applied potential, *V*_FB_ is the flat-band potential, *e* is the charge of the electron, and *N*_d_ is the donor density. The depletion layer width for the binary Fe_2_O_3_ was found to be 28 nm, which is larger than that of the doped sample (21 nm).

### 3.4 Characterization of the Optimized Photoelectrode

**Figure 12:**
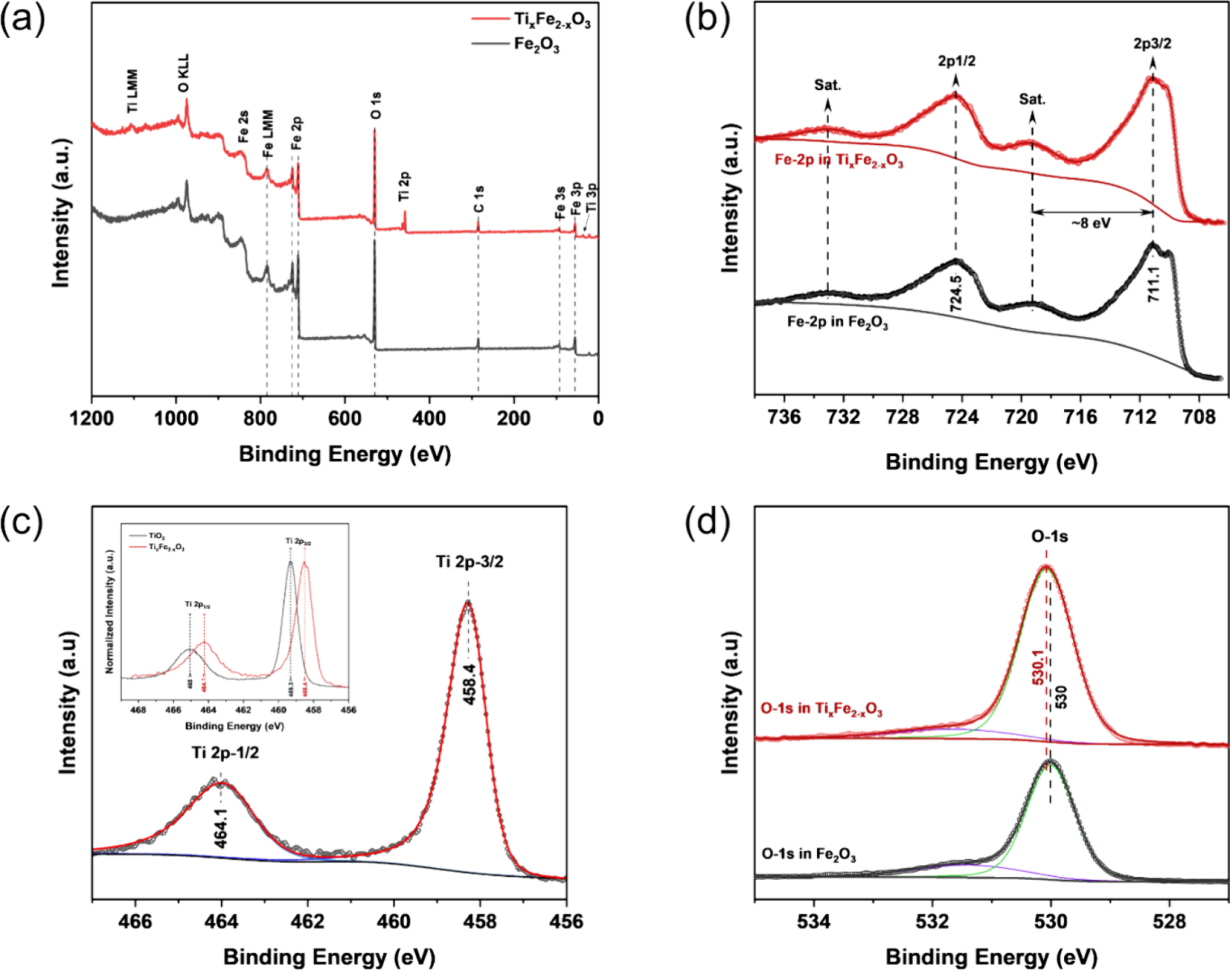
(a) XPS survey spectra of Fe_2_O_3_ and Ti_*x*_Fe_2–*x*_O_3_, high-resolution XPS spectrum of (b) Fe 2p, (c) Ti 2p (inset: Ti 2p in TiO_2_ and Ti*x*Fe_2–*x*_O_3_) and (d) O 1s.

The oxidation states of Fe and Ti in both Fe_2_O_3_ and Ti_*x*_Fe_2–*x*_O_3_ photoelectrodes were determined by XPS analysis. The survey scan clearly demonstrates the presence of Ti in the doped sample (Figure 12a). The high-resolution Fe 2p XPS spectra (Figure 12b) of both the undoped and doped films demonstrate the Fe 2p_3/2_ and Fe 2p_1/2_ peaks with their accompanied satellite peaks, which is a fingerprint of the Fe^3+^ state. The main peaks were located at binding energies of 711.1 and 724.5 eV, and the corresponding satellite peaks were at around 8 eV higher binding energies. The absence of the Fe^2+^ peak at 716 eV and the presence of satellite peaks without any noticeable decrease in intensity with the Ti-doping confirm that Fe exists in the Fe^3+^ form in both the undoped and doped samples.

Figure 12c shows a high-resolution Ti 2p spectrum of the doped sample with two peaks of Ti 2p3/2 and Ti 2p_1/2_ centered at binding energies of 458.4 and 464.1 eV, respectively. These peaks indicate the presence of Ti^4+^ valence state in the Fe_2_O_3_ matrix. Notably, a binding energy shift of approximately 0.9 eV is observed in the Ti_*x*_Fe_2–*x*_O_3_ sample compared to TiO_2_ (inset Figure 12c). This shift suggests a different electronic environment of Ti in Ti_*x*_Fe_2–*x*_O_3_ compared to TiO_2_ and implies that Ti atoms are likely incorporated into the crystal structure of Fe_2_O_3_. Typically, the hematite lattice features a hexagonally close-packed arrangement of O^2–^ ions, with the Fe^3+^ ions occupying two-thirds of the available octahedral sites. Ti^4+^ ions and other cationic dopants can substitute Fe^3+^ ions at these octahedral sites or occupy interstitial positions.^56,57^ Our investigation, which involves a relatively high concentration of Ti^4+^ and the absence of Fe^2+^ ions, suggests that three Ti^4+^ ions are incorporated as ‘defect clusters’ at both substitutional and interstitial octahedral sites. This incorporation of three Ti^4+^ cations is accompanied by the removal of four Fe^3+^ ions, serving as a charge compensation mechanism.

The high-resolution O 1s spectra (Figure 12d) can be deconvoluted into two peaks: lattice oxygen (O_L_) and adsorbed oxygen (O_A_). The intense peak located at 530.1 eV represents the oxygen ion in the crystal lattice, while the broad peak at around 531.5 eV may be attributed to the adsorbed oxygen species, brought on by oxygen antisite (O_Fe_), oxygen vacancy, and oxygen interstitial.^58,59^

**Figure 13:**
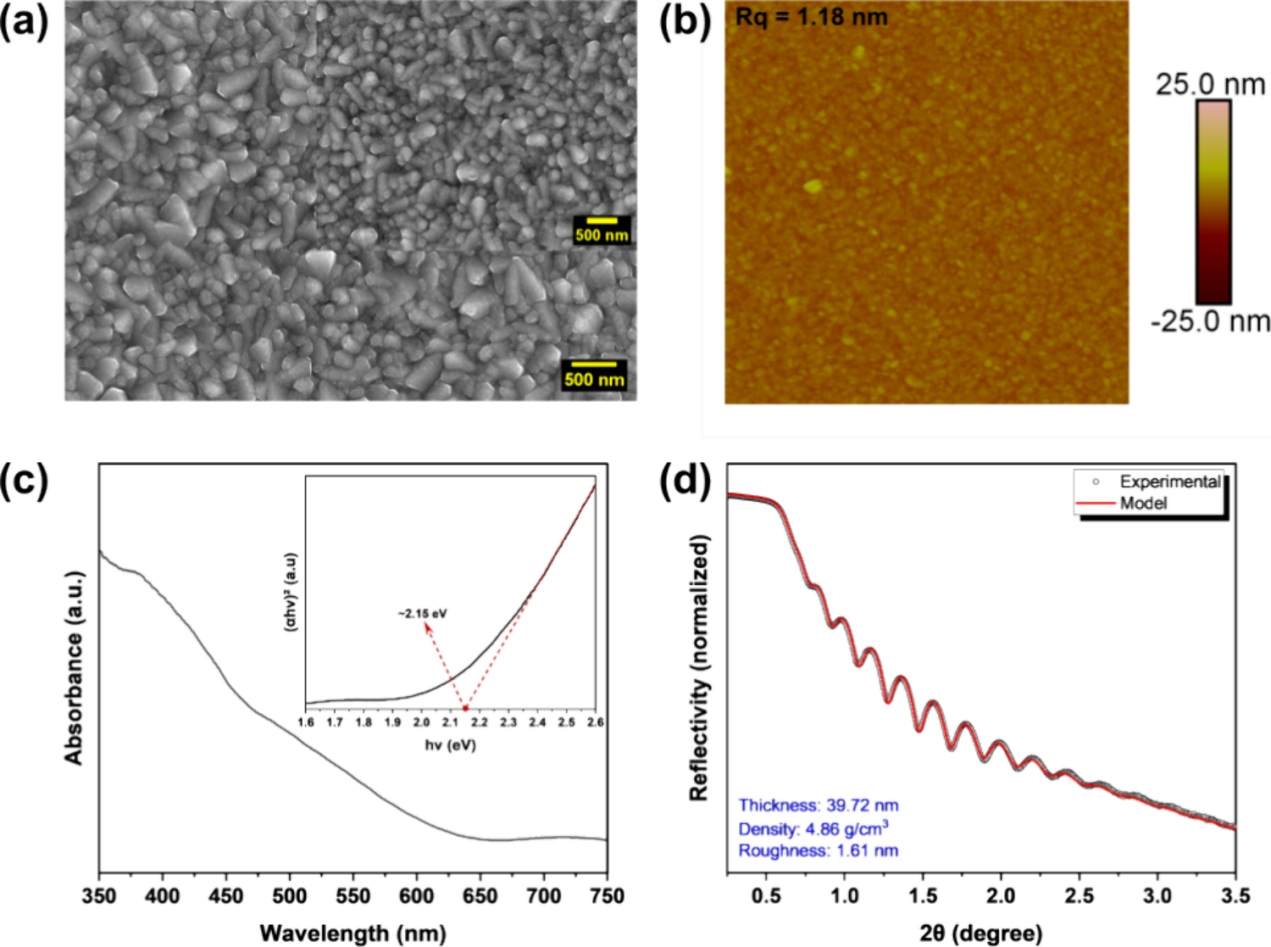
(a) FESEM image of the optimized Ti_*x*_Fe_2–*x*_O_3_ photoanode grown on FTO (inset: FTO substrate), (b) AFM image on the Si (c) UV–vis absorption spectrum of the Ti_*x*_Fe_2–*x*_O_3_ film grown on FTO (inset: Tauc plot obtained form the absorption spectrum) (d) X-ray reflectivity profile of Ti_*x*_Fe_2–*x*_O_3_ grown on Si.

A top-down FESEM image of the optimized Ti_*x*_Fe_2–*x*_O_3_ film grown on the FTO substrate is shown in Figure 13a, with the inset displaying the surface morphology of the bare FTO. The deposition of ∼40 nm Ti_*x*_Fe_2–*x*_O_3_ film on the FTO substrate preserves the underlying texture of the bare FTO, indicating uniform and conformal growth. Additionally, an AFM image of Ti_*x*_Fe_2–*x*_O_3_ deposited on a Si substrate shows polycrystalline morphology, characterized by a surface roughness of 1.2 nm (Figure 13b). Figure 13c illustrates an absorbance spectrum of the optimized Ti_*x*_Fe_2–*x*_O_3_ photoelectrode, with corrections applied for both the reflectance and absorbance of the FTO substrate, following methodologies detailed in existing literature.^60^ The inset presents the corresponding Tauc plot, which indicates a band gap energy of 2.15 eV, aligning well with the previously reported value.^25,36^Figure 13d depicts the X-ray reflectance (XRR) pattern of the optimized Ti_*x*_Fe_2–*x*_O_3_ photoelectrode. The experimental results were modeled to derive film thickness, density, and surface roughness, resulting in a well-correlated fit. The film thickness was determined to be 39.7 nm, which closely aligns with the thickness (39.4 nm) measured with EDS on FTO. The film density was found to be 4.86 g/cm^3^ and the surface roughness 1.61 nm, as extracted from the XRR fit. The roughness value obtained from the XRR analysis closely aligns with the roughness value (1.18 nm) obtained from the AFM measurements.

## 4 Conclusions

In this study, RSM-based experimental design was employed for modeling and optimization of the photocurrent response of Ti_*x*_Fe_2–*x*_O_3_ photoanodes grown by the ALD technique. The FC-CCD based experimental design was adopted to study the effects of the dopant concentration, film thickness, and deposition temperature on the photocurrent response of the Ti_*x*_Fe_2–*x*_O_3_ photoanodes. Analysis of experimental data suggests that film thickness and dopant concentration are the most significant factors influencing the photocurrent response, as confirmed by high correlation between the predicted and experimental data. The FC-CCD model predicts that an optimum photocurrent response at 1.23 and 1.70 V can be achieved with 490 deposition cycles with a TiO_2_ to Fe_2_O_3_ cycle ratio of 1/4 at a deposition temperature of 275 °C. The model was validated through the fabrication of a Ti_*x*_Fe_2–*x*_O_3_ photoanode using the optimized deposition condition and by testing its performance. This work emphasizes the effectiveness of RSM-based modeling in predicting and optimizing photoelectrode performance, providing valuable insights into the design of hematite-based photoanodes for advanced photoelectrocatalytic applications.

## Supporting Information Available

Figures associated with the ALD growth characteristics of Ti_*x*_Fe_2–*x*_O_3_ (Figure S1), FESEM images (Figure S2), LSV profiles of Ti_*x*_Fe_2–*x*_O_3_ photoanodes (Figure S3), Tables for the FC-CCD experimental design matrix (Table S1), and model parameter estimates (Tables S2 and S3) (PDF)
