# Supplementary material for: Atomic Layer Deposition of TixFe2–xO3 Photoanodes and Photocurrent Response Optimization Using the Response Surface Methodology
Source: ACS Omega. 2025 Apr 5;10(14):14522–35. doi: 10.1021/acsomega.5c01360 (PMC12004141; doi:10.1021/acsomega.5c01360)
Supplement: Supplementary file 1 — ao5c01360_si_001.pdf [file ao5c01360_si_001.pdf]

Supplementary Files

Atomic Layer Deposition of $Ti_xFe_{2-x}O_3$ Photoanodes and Photocurrent Response Optimization using Response Surface Methodology

Anjan Deb*, Anton Vihervaara, Georgi Popov, Mykhailo Chundak, Ahmed O. Abdelaal, Hugo L. S. Santos, Mikko J. Heikkilä, Marianna Kemell, Pedro H. C. Camargo, Mikko Ritala and Matti Putkonen*

Department of Chemistry, University of Helsinki, P.O. Box 55, FI-00014 Helsinki, Finland.

*Corresponding author: anjan.deb@helsinki.fi, matti.putkonen@helsinki.fi

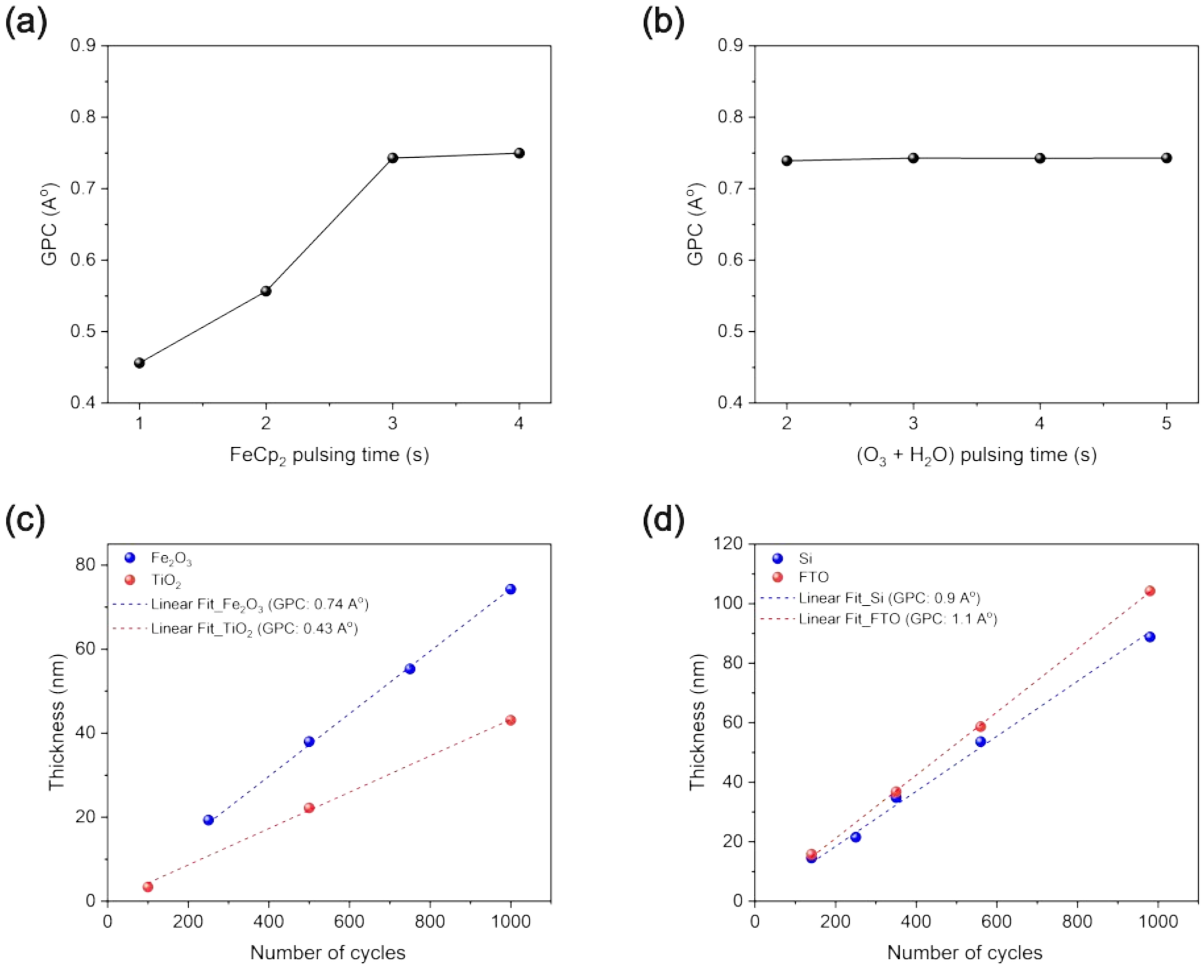

Fig. S 1: Growth of Fe_2O_3 on Si for 1000 cycles at 300°C as a function of (a) $FeCp_2$ pulsing (b) O_2+H_2O pulsing (c) Growth of Fe_2O_3 and TiO_2 on Si as a function of cycle numbers at 300°C. (d) Growth of $Ti_xFe_{2-x}O_3$ (Ti:Fe = 1:4) on Si and FTO as a function of cycle number at 300°C. All the thickness were calculated from the k-ratio of EDS measurement using the densities of 5.25 and 4.23 g/cm^3 for Fe_2O_3 and TiO_2, respectively.

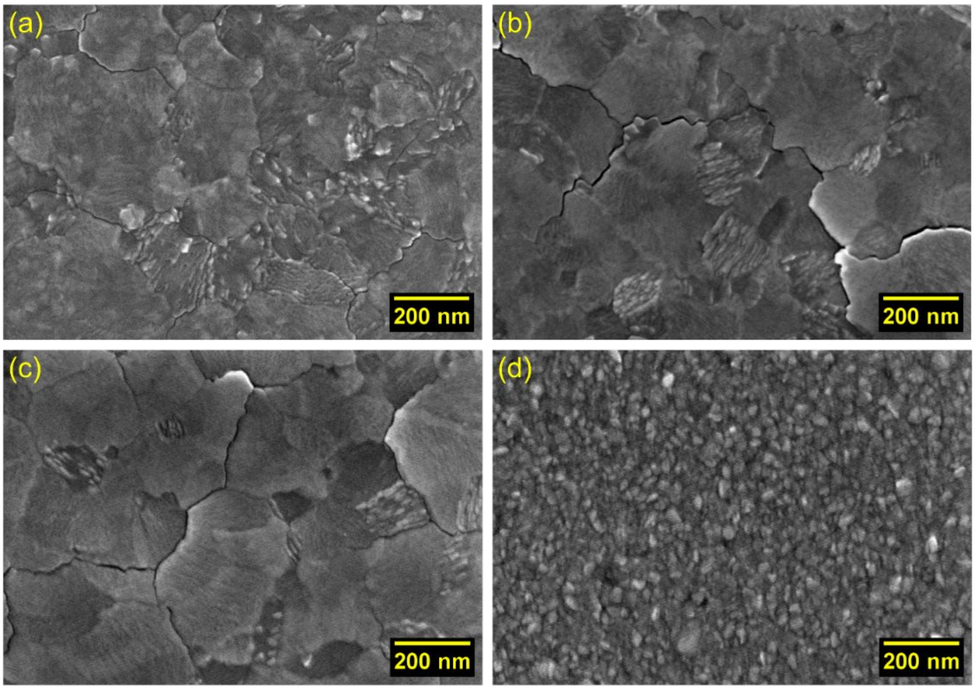

Fig. S 2: FESEM images of (a) Fe_2O_3 (b-d) $Ti_xFe_{2-x}O_3$ with Ti:Fe 1/34, 1/19 and 1/4, respectively. All the films are deposited at 300°C on Si substrate.

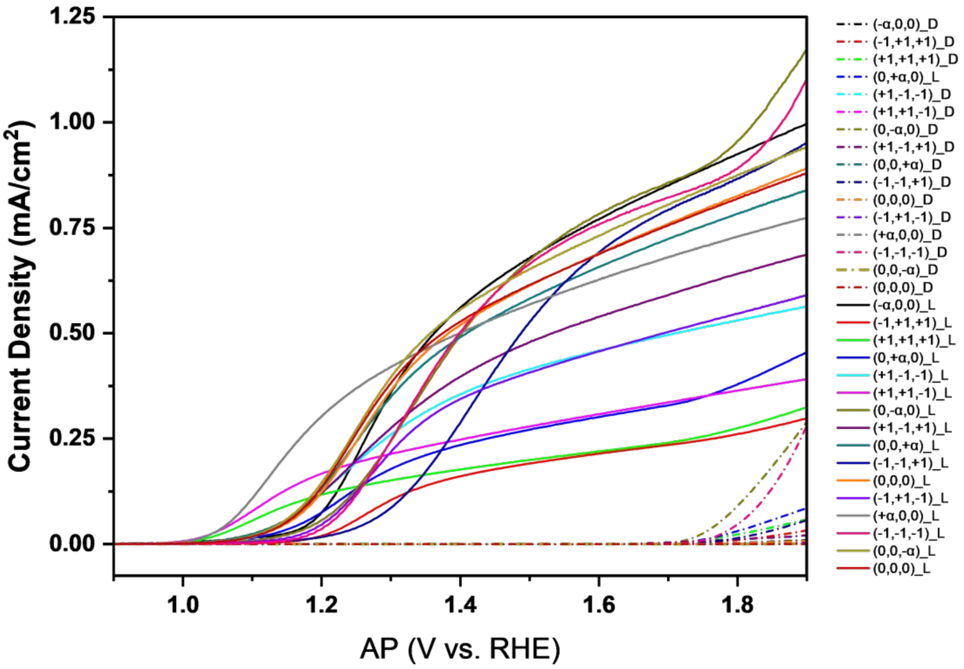

Fig. S 3: Linear Sweep Voltametric (LSV) profiles of $Ti_xFe_{2-x}O_3$ photoanodes.

Table S 1: Experimental Design Matrix based on FC-CCD

**No of Exp.**		**Coded factors**			**Decoded factors**			**Responses**		**Other parameters**	
		CR	TC	DT	CR	TC	DT	PCD @1.23V vs RHE (mA/cm²)	PCD @1.7V vs RHE (mA/cm²)	Film thickness (nm)	Dopant Conc. (%)
**1**	Factorial runs, 2^k	-1	-1	-1	1/34	140	250	0.08	0.82	9.8±0.6	n/d
**2**		+1	-1	-1	1/4	140	250	0.17	0.50	8.5±0.5	11.4±0.2
**3**		-1	+1	-1	1/34	980	250	0.09	0.50	58.1±0.6	2.1±0.2
**4**		+1	+1	-1	1/4	980	250	0.18	0.34	64.1±0.5	18.1±0.4
**5**		-1	-1	+1	1/34	140	300	0.03	0.79	13.3±0.4	n/d
**6**		+1	-1	+1	1/4	140	300	0.17	0.59	15.8±0.4	13.7±0.5
**7**		-1	+1	+1	1/34	980	300	0.04	0.24	110±0.6	1.9±0.4
**8**		+1	+1	+1	1/4	980	300	0.13	0.24	104.3±0.7	18.4±0.2
**9**	Axial point runs, 2k	-α	0	0	1/34	560	275	0.15	0.85	41.7±1.3	2.7±0.1
**10**		+α	0	0	1/4	560	275	0.35	0.68	46.2±0.8	15.2±0.8
**11**		0	-α	0	1/19	140	275	0.10	0.86	13.1±0.6	n/d
**12**		0	+α	0	1/19	980	275	0.11	0.33	91.9±1.4	3.6±0.7
**13**		0	0	-α	1/19	560	250	0.22	0.81	38.4±0.7	5.0±0.5
**14**		0	0	+α	1/19	560	300	0.20	0.72	49.7±1.5	3.5±0.3
**15**	Center points, C_o	0	0	0	1/19	560	275	0.20	0.76	46.1±0.1	3.6±0.3
**16**		0	0	0	1/19	560	275	0.21	0.76	46.1±0.4	3.6±0.4

n/d = non-detectable

Table S 2: Parameter estimates and effect tests @ 1.23V

Term	Estimate	Std. Error	t Ratio	Sum of Squares	F Ratio	Prob>|t|
Intercept	0.216	0.013	16.22			<.0001*
CR	0.061	0.009	6.85	0.0373	46.99	0.0005*
TC	0.001	0.009	0.15	0.0000	0.02	0.8864
DT	-0.017	0.009	-1.95	0.0030	3.79	0.0994
CR*TC	-0.005	0.010	-0.55	0.0002	0.30	0.6014
CR*DT	0.006	0.010	0.57	0.0003	0.32	0.5906
TC*DT	-0.007	0.010	-0.70	0.0004	0.49	0.5086
CR*CR	0.026	0.017	1.48	0.0017	2.19	0.1896
TC*TC	-0.118	0.017	-6.82	0.0369	46.47	0.0005*
DT*DT	-0.012	0.017	-0.66	0.0004	0.44	0.5314

Table S 3: Parameter estimates and effect tests @ 1.70V

Term	Estimate	Std. Error	t Ratio	Sum of Squares	F Ratio	Prob>|t|
Intercept	0.787	0.025	31.66			<.0001*
CR	-0.086	0.017	-5.18	0.074	26.81	0.0021*
TC	-0.192	0.017	-11.53	0.367	132.99	<.0001*
DT	-0.038	0.017	-2.28	0.014	5.20	0.0627
CR*TC	0.046	0.019	2.47	0.017	6.10	0.0485*
CR*DT	0.037	0.019	1.99	0.011	3.97	0.0935
TC*DT	-0.054	0.019	-2.90	0.023	8.40	0.0274*
CR*CR	-0.036	0.032	-1.11	0.003	1.23	0.3094
TC*TC	-0.208	0.032	-6.44	0.114	41.43	0.0007*
DT*DT	-0.037	0.032	-1.15	0.004	1.32	0.2949
